# A novel approach for superficial intraoperative radiotherapy (IORT) using a 50 kV X‐ray source: a technical and case report

**DOI:** 10.1120/jacmp.v15i1.4502

**Published:** 2014-01-06

**Authors:** Frank Schneider, Sven Clausen, Johannes Thölking, Frederik Wenz, Yasser Abo‐Madyan

**Affiliations:** ^1^ Department of Radiation Oncology University Medical Center Mannheim, University of Heidelberg Mannheim Germany; ^2^ Department of Radiation Oncology and Nuclear Medicine Faculty of Medicine, Cairo University Cairo Egypt

**Keywords:** intraoperative radiotherapy, electronic brachytherapy, X‐ray, INTRABEAM radiotherapy system, superficial radiotherapy

## Abstract

The use of IORT as a treatment modality for patients with close or positive margins has increased over the past decade. For situations where a flat area (up to 6 cm in diameter) has to be treated intraoperatively, new applicators for superficial treatment with a miniature X‐ray source (INTRABEAM system) were developed. Here we report our evaluation of the dosimetric characteristics of these new applicators and their first clinical use. Each of these flat and surface applicators consists of a radiation protective metal tube and a flattening filter, which converts the spherical dose distribution of the X‐ray source into a flat one. The homogeneity of each dose distribution and depth‐dose measurements were evaluated using film dosimetry in a solid water phantom and a soft X‐ray ionization chamber in a water tank. The first patient was treated with 5 Gy delivered in 5 mm using a 4 cm FLAT applicator over 21 minutes. The flat applicators show the maximum homogeneity, with a uniformity ratio of 1.02‐1.08 in certain depths. In 1 mm depth surface applicators show a uniformity ratio of 1.15‐1.28. They also show a higher dose rate and a steeper dose gradient compared to the flat applicators. The results of this investigation demonstrated that the flat and surface applicators have unique dosimetric characteristics that need to be considered during the treatment planning stages. This work also showed that it is possible to perform a superficial localized IORT which provides new application possibilities for use of the INTRABEAM system.

PACS number: 87.55.ne

## I. INTRODUCTION

The use of intraoperative radiotherapy (IORT) as a treatment modality for patients with close or positive margins has increased over the past decade. It is commonly done with high voltage electrons,^1^, ^2^ HDR brachytherapy^3^, ^4^, ^5^ or particularly with low kV X‐ray IORT.^6^, ^7^, ^8^, ^9^ Low kV sources are of interest because of minimal radiation protection requirements.^10^, ^11^ The XRS 4 (INTRABEAM System) has been mainly used for intraoperative tumor bed treatment of breast cancer^(6)^ and spinal metastasis^12^, ^13^, ^14^ using dedicated spherical or needle applicators. Retroperitoneal cancer, such as sarcomas, can also benefit from IORT^(15)^ because postoperative external beam radiotherapy (EBRT) is limited by the tolerance of the surrounding healthy tissues.^(16)^ Hu and Harrison^(17)^ summarize an improvement in local control with an IORT to the flat tumor bed using HDR brachytherapy with a Harrison‐Anderson‐Mick (HAM) applicator or high‐energy electron treatment with an electron cone. Similar effects are discussed by Dubois et al.^(18)^ who performed IORT of locally advanced or recurrent rectal cancer with high‐energy electrons to a flat target volume with a diameter of 5‐10 cm and a thickness of 1‐5 cm. However, using HDR brachytherapy or high‐energy electrons requires substantial radiation protection measure, such as a specially shielded operating room. The patient has to be transported to or operated in that OR, and the staff is not allowed to stay in the room during radiotherapy. Alternatively, low kV X‐rays can be used, as described in Guo et al.^(19)^ They described comparable rates of toxicity, local recurrence, and survival rates treating locally advanced or recurrent rectal cancer with the INTRABEAM system. They treated areas of concern for a close or involved radial margin of initial tumor sizes of 0‐10 cm. To get an almost flat dose distribution, they chose mostly a 5 cm spherical applicator (prescription of 5 Gy to a depth of 1 cm). Small lead sheets were used to protect the surrounding tissue.

Those studies demonstrated that IORT is beneficial for many treatment sites, but an applicator is needed to treat flat areas. Thus new applicators for superficial treatment with the INTRABEAM system were developed. Here we report our evaluation of the dosimetric characteristics, such as homogeneity of the dose distribution and the depth dose, of these new applicators and their first clinical use.

## II. MATERIALS AND METHODS

### A. characteristics of the superficial applicators

The INTRABEAM system (Carl Zeiss Meditec AG, Oberkochen, Germany) consists of the user terminal (the graphic interface between user and control console); the control console, which controls the X‐ray source (XRS 4); quality assurance (QA) equipment; and the XRS 4. Additionally, a support stand and different applicators are commercially available. The XRS 4 itself consists of an electron gun, which emits electrons; the accelerating unit, which accelerates the electrons to a maximum of 50 kV; and two pairs of bending coils, which guide the electron beam through a 10 cm long field‐free drift tube (probe) to the gold target, where Bremsstrahlung is generated. This results in a spherical dose distribution.^11^, ^20^

To treat small flat areas in intraoperative situations, applicators which transform the spherical dose distribution into a flat circular one and which shield the surrounding tissue were designed in collaboration between Carl Zeiss Meditec AG and our center. Those now commercially available applicators consist of the main body, a metal tube, with a 0.05 mm Pb‐equivalent shielding effect which attenuates more than 90% of the dose as described in prior measurements using the same X‐ray source and attenuation material with the same Pb equivalence;^(10)^ and the flattening filter, an absorption body which is fixed with a metal cap. The flattening filters, made of polyetherimide (PEI), the same amber‐to‐transparent thermoplastic (mass density of $1.27\;g\;{\text{cm}}^{-3}$) that is used for the spherical applicators, were designed to create a homogeneous dose distribution at the surface of the applicator (surface applicator) or in a certain depth (flat applicator). Depending on the type and diameter of the applicators, the thickness of the filters and the source‐to‐surface distance of the applicators vary. The surface applicators, available in diameters of 1, 2, 3, and 4 cm, have a source‐to‐surface distance of 9.6‐21.6 mm, with a filter thickness of 2.7‐5.4 mm. The flat applicators, available in diameters of 1, 2, 3, 4, 5, and 6 cm, have a source‐to‐surface distance of 9.6‐25.6 mm, with a filter thickness of 7.0‐23.5 mm. To mark the region of interest in the tumor bed or to inhibit the movement of the applicator relative to the target (e.g., caused by breathing), a stainless steel ring (positioning marker) can be stitched in the wound cavity.

### B. Dose distribution

**Figure 1:**
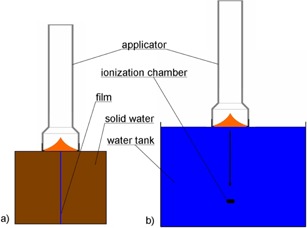
Schematic setup to measure: (a) dose distribution in different depths with films, and (b) dose rate dependence on depth measured in water with a soft X‐ray ionization chamber.

To verify the different dose distributions, GAFCHROMIC EBT2 films/film pieces (ISP, NJ) were sandwiched between two solid water blocks (Gammex 457; Gammex Inc., Middleton, WI). The applicator was attached to the XRS 4 and the support stand, and it was aligned with the applicator touching the surface of the phantom with the center axis of the applicator in line with the film (see Fig. 1(a)).

**Figure 2:**
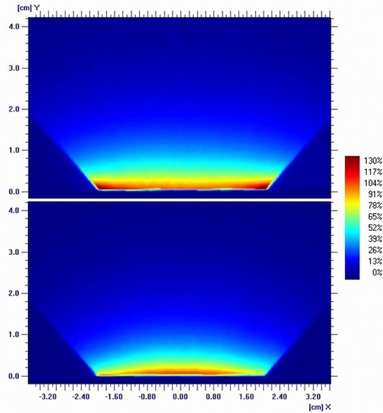
Normalized dose distribution (normalization depth: 1 mm) of a 4 cm flat (top) and a 4 cm surface applicator (bottom).

The irradiated films were scanned (resolution 72 ppi) with an EPSON Expression 10000XL/PRO flatbed scanner (US Epson, Long Beach, CA) after a developing time of 12 hours in “Portrait” mode. To guarantee that the films/film pieces lay flat in the center of the scanner plate, a specially designed plastic frame, the EASEL (ISP, NJ), and lead coins placed at the corners of the film, were used. The 16 bit “red” images of the 48 bit RGB scans (without any corrections) were calibrated (grey values into absolute dose) using a previously developed calibration curve^(11)^ and evaluated in OmniPro‐I'mRT 1.6 (IBA Dosimetry GmbH, Schwarzenbruck, Germany) (Fig. 2). Uniformity ratios, maximum dose (Dmax) divided by minimum dose (Dmin), were calculated from 90% of the field size in the depths of 1 mm, 5 mm, and maximum homogeneity (dhmax) for the flat applicators, and in the depths of 1 mm and 5 mm for the surface applicators. The individual field size in each depth was defined at 50% of the center axis dose of the profile.

### C. Depth dose

Depth‐dose measurements were completed with the XRS 4 and applicator mounted to a dedicated holder of a water tank (Carl Zeiss Surgical GmbH). A soft X‐ray ionization chamber (volume: 0.0053 cm^3^, type 34013, Physikalisch‐Technische Werkstaetten, Freiburg, Germany) was placed in the dedicated solid water holder of the phantom. The XRS 4 with the applicator was then moved in steps of 0.5 mm along the center axis (see Fig. 1(b)).

## III. RESULTS

### A. Dose distribution

**Figure 3:**
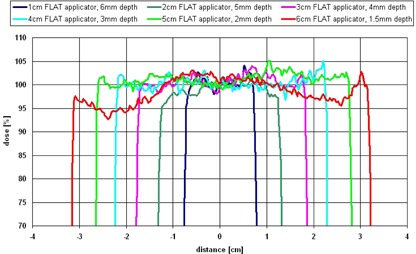
Profiles of the flat applicators (1‐6 cm) in dhmax, center axis dose normalized to 100%. Due to the divergence of the beam, the displayed field sizes are bigger than the applicator diameter (field size at the surface of the applicator).

The evaluation of the films of the flat applicators showed that the dhmax depends on applicator size. These depths range from 6 mm (size: 1 cm) down to 1.5 mm (size: 6 cm). Normalized profiles in dhmax are shown in Fig. 3 and the calculated uniformity ratios in the depth of 1 mm, dhmax, and 5 mm are listed in Table 1.

Because of resolution and scanner artifacts the film edge of the surface applicator measurement couldn't be evaluated. However, to get an idea of the homogeneity, profiles were taken in depths of 1 mm and 5 mm. The calculated uniformity ratios are listed in Table 2.

**Table 1:** Uniformity of flat applicator profiles at depths of maximum homogeneity (dhmax), 1 mm and 5 mm

*Size of Flat Applicator*	*dhmax (mm)*	*Uniformity in 1 mm*	*Uniformity in dhmax*	*Uniformity in 5 mm*
1 cm	6.0	1.25	1.03	1.05
2 cm	5.0	1.26	1.02	1.02
3 cm	4.0	1.31	1.03	1.08
4 cm	3.0	1.31	1.02	1.13
5 cm	2.0	1.15	1.04	1.20
6 cm	1.5	1.09	1.08	1.23

**Table 2:** Uniformity of surface applicator profiles at depths of 1 mm and 5 mm

*Size of Surface Applicator*	*Uniformity in 1 mm*	*Uniformity in 5 mm*
1 cm	1.15	1.21
2 cm	1.13	1.25
3 cm	1.20	1.40
4 cm	1.28	1.47

### B. Depth dose

**Figure 4:**
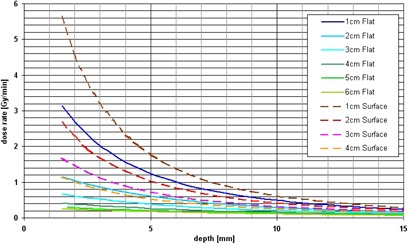
Dose rate dependence on depth of the flat (solid lines) and surface applicators (dashed lines) measured in a water tank.

The dose‐rate dependence on depth measured in the water tank is shown in Fig. 4. Because of the distance of 1.5 mm from the solid water holder surface to the effective point of measurement of the ionization chamber, the curves start at 1.5 mm.

For the surface applicators a thinner flattening filter was designed, which results in a higher dose rate of $1.1-5.65\;{\text{Gy min}}^{-1}$ in 1.5 mm depth compared to $0.4-3.15\;{\text{Gy min}}^{-1}$ for the equivalent flat applicator sizes (1‐4 cm). This shortens the treatment time, but decreases the homogeneity in the target volume because of the steeper dose gradient.

Prescribing 5 Gy in 5 mm tissue depth the treatment, using the flat applicators (1‐6 cm), would last 5‐30 min. To treat with 5 Gy at the surface of the surface applicators (1‐4 cm), the dose rate curves can be extrapolated, resulting in a treatment time of approximately 0.5‐3 min.

#### Case Report

**Figure 5:**
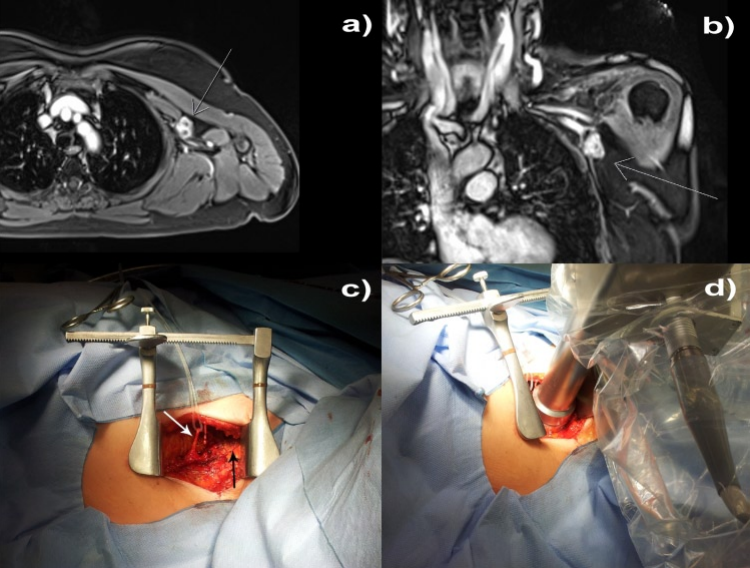
Intraoperative treatment: (a) axial and (b) coronal magnetic resonance images showing the relationship between the 3 cm irregular auxiliary mass to the auxiliary vessel/nerve complex; (c) the auxiliary vessels exposed (black arrow), thoracodorsal nerve placed aside (white arrow); and (d) the applicator setup.

A 70‐year‐old female presented to our department with a 3 cm irregular left auxiliary mass. Diagnostic ultrasound, computer tomography, and magnetic resonance imaging revealed direct contact with the auxiliary vessels and the auxiliary nerves (Figs. 5(a) and (b)). The patient's medical history included invasive ductal cancer of the left breast nine years earlier (initially cT2 cN0 M0) treated with neoadjuvant chemotherapy (6x DAC), breast conserving surgery, auxiliary lymph node dissection (ypT1b ypN0 R0 G2 ER+PR+Her2neu 0), adjuvant radiotherapy to the left breast and lower axilla (without the auxiliary apex or the supraclavicular region) up to 50 Gy, followed by adjuvant hormonal treatment. The current auxiliary mass was located at the upper edge of the previously irradiated volume. An ultrasound‐guided auxiliary biopsy revealed a malignant sarcomatous growth, which based its location at the inner edge of the old radiation field used to treat the patient for breast cancer, and the latency period of nine years fits the pattern of a radiation‐induced secondary cancer.^21^, ^22^, ^23^, ^24^ Surgical dissection of the auxiliary mass was planned. Given the expected difficulty to achieve wide resection margins close to the auxiliary vessels and the added difficulty of postoperatively reirradiating the axilla to an adequate dose, intraoperative irradiation of the tumor bed, including the vascular sheath, was proposed in case inadequate resection margins were found. Patient's consent was obtained. Macroscopically removed, the attachment of the mass to the sheath of the auxiliary vessels prevented the surgeon from reaching clear resection margins. The decision to use IORT was made intraoperatively, based on the judgment of the experienced surgeon and the radiation oncologist. To reduce the risk of local auxiliary recurrence, intraoperative irradiation of the vascular sheath and the surrounding auxiliary fat at the resection bed was performed using the 4 cm flat applicator, which was optimal in regard to shape and size to cover the area to be treated. The thoracodorsal nerve was dissected and displaced temporarily outside of the radiation field (Figs. 5(c) and (d)). The brachial plexus was located 1.5‐2 cm deep to the applicator and required no special protection measures. A dose of 5 Gy was delivered in 5 mm depth (corresponds to 6.5 Gy at 3 mm (dhmax), 10.5 Gy at the applicator surface and 1.7 Gy in 15 mm depth (location of the brachial plexus)) over 21 minutes. The IORT dose was chosen to be used as an early boost within the total concept of adjuvant radiotherapy that included a planned postoperative external beam radiotherapy (EBRT) to the axilla and, after considering the previous exposure of the brachial plexus nine years ago, estimated to be equivalent to 24 Gy in 2 Gy fractions. Histopathological and immunohistochemical examinations of the removed mass revealed a pT2b pN0 M0 G3 angiosarcoma. The patient was discharged from hospital on the third day after surgery to regular outpatient postoperative follow‐up. No treatment‐related side effects were observed five months after radiotherapy.

## IV. DISCUSSION

Film dosimetry is a common tool in evaluation of dose distributions in radiotherapy.^25^, ^26^ Contrary to many advantages of using GAFCHROMIC films (such as it may be handled in visible light, there is no variation because of wet processor performance, and better tissue equivalence than radiographic film), one disadvantage is the nonlinearity of pixel value (grey value) to dose.^27^, ^28^, ^29^ In order to evaluate the dose homogeneity, we had to convert pixel values into absolute dose. This calibration curve, developed previously,^(11)^ was based on absolute dose measurements using a soft X‐ray ionization chamber (Type 23342, Physikalisch‐Technische Werkstaetten), which has a bigger sensitive volume (0.02 cm^3^) than the chamber used in that study to measure the depth dose (0.0053 cm^3^). This volume difference influences the depth dose up to 30% (depending on applicator and size). This will affect the ratio of pixel value to dose of the calibration which could slightly affect the ratio of center dose to edge dose of the profiles, which would influence the uniformity ratios (max. 4% for the worst uniformity: 4 cm surface applicator in 5 mm depth). However, due to the nonlinearity of pixel value to dose, a relative comparison of the profiles (without absolute calibration) would lead to an underestimation of the uniformity ratio up to 30%.

In this report, we made the assumption that the dose distribution is radial symmetric because of the manufacturing tolerances of the applicator (main body and flattening filter), which allows a reproducible position of source to applicator. However, the source itself shows a deviation of <3% in the radial plane, which will also slightly influence the uniformity values estimated based on profile measurements.

The absolute dosimetry with ionization chambers depends on the beam quality/the energy spectrum of the system. With decreasing the flattening filter thickness, the mean energy of the system is also decreasing. This was shown by Eaton and Duck^(30)^ who evaluated the half value layers (HVLs) for the INTRABEAM system with different spherical applicators, which are made of the same material as the flattening filters. They evaluated HVLs of 0.85‐1.3 mm Al and compared it to a reference value of 0.64 mm Al, as measured by Zeiss using a 1 cm solid water buildup. For the surface applicators, the filters are even thinner, which further decreases the HVL/mean energy. However, this variance in mean energy results in a maximum difference in the beam quality factor of 3.6%, which is minor compared to the variances caused by geometrical misplacements (about $10\%–40\%\;{\text{mm}}^{-1}$ depending on applicator size and treatment depth).

**Figure 6:**
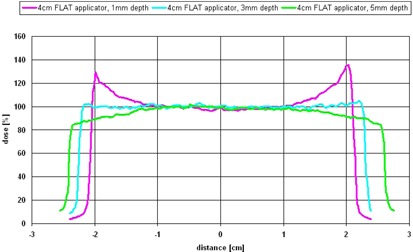
Uniformity of a 4 cm flat applicator in 1 mm (top line), dhmax=3 mm (middle line), and 5 mm (bottom line) depths, with center axis dose normalized to 100%.

The flat applicators each demonstrate a unique depth at which the maximum homogeneity was observed. To get a small uniformity ratio (<1.03 along the profile) in the same depth (e.g., 5 mm) for all applicators, the flattening filter must be changed. But this would also influence the uniformity at the surface of the applicator. For example, a 4 cm flat applicator actually shows the minimum uniformity ratio in the dhmax of 3 mm depth. The edge dose in 5 mm depth is 13% lower, while the edge dose in 1 mm depth is 31% higher than the central axis dose (see Fig. 6). If the flattening filter would be changed to have a minimum uniformity in 5 mm depth, the edge dose at the surface of the applicator would be even larger.

A challenge is to evaluate the absolute dose and the uniformity at the surface of the applicators. If a film is used as shown in this report, the artifacts based on the scanner and the resolution do not make it possible to evaluate the first millimeter of the film. Even if the film is positioned horizontally (parallel with the applicator surface), the minimum distance from the surface of the applicator to the active layer of the film is 0.2 mm. If a soft X‐ray ionization chamber is used to measure the absolute dose and the homogeneity in a water tank, a solid water holder has to be used which inhibits measurements close to the surface. However, to evaluate only the absolute dose, the chamber can be used in a solid water phantom (sensitive volume in plane with the phantom surface),^(20)^ such as Gammex RMI457 which has the best water equivalency using low kilovolt X‐rays.^(31)^ Further research is needed to investigate the uniformity at the surface of the applicators, perhaps by Monte Carlo simulations, as shown by Clausen et al.^(32)^ and Nwankwo et al.^(33)^

## V. CONCLUSIONS

Many previous studies (e.g., Guo et al.^(19)^), described that a superficial kV IORT is beneficial and needed for many treatment sites. The present work shows that the INTRABEAM system using the newly developed flat or surface applicators can be used for those paradigms. The reported uniformities and dose distributions were judged to be clinically acceptable for the case described. The provided data, which helps the clinician and medical physicist to choose the approach for the individual patient, and the additional shielding effect of the applicator's main body, provides new application possibilities for use of the INTRABEAM system.

## Supporting information

Supplementary Material
