# Supplementary material for: A novel approach for superficial intraoperative radiotherapy (IORT) using a 50 kV X‐ray source: a technical and case report
Source: J Appl Clin Med Phys. 2014 Jan 6;15(1):167–76. doi: 10.1120/jacmp.v15i1.4502 (PMC5711231; doi:10.1120/jacmp.v15i1.4502)
Supplement: Supplementary file 1 — Supplementary Material [file ACM2-15-167-s001.doc]

A novel approach for superficial intraoperative radiotherapy (IORT) using a 50 kV x-ray source: A technical and case report

**Frank Schneider (PhD), Sven Clausen (PhD), Johannes Thölking (MSc),**

**Frederik Wenz (MD), Yasser Abo-Madyan (MD)**

Department of Radiation Oncology

University Medical Center Mannheim, University of Heidelberg

*Theodor-Kutzer-Ufer 1-3, 68167 Mannheim, Germany*

*Frank.schneider@umm.de, sven.clausen@umm.de, Johannes.Thoelking@umm.de, frederik.wenz@medma.uni-heidelberg.de, yasser.abomadyan@umm.de*

**Yasser Abo-Madyan (MD)**

Department of Radiation Oncology and Nuclear Medicine

Faculty of Medicine, Cairo University

*Al-Saray Street, El Manial, Cairo 11956, Egypt*

*yasser.abomadyan@umm.de*

Running title: Superficial intraoperative radiotherapy
